# Supplementary material for: Cervical mucus viscoelasticity and sperm velocity are correlated and concentration-dependent in Vitro
Source: PLoS One. 2026 Apr 7;21(4):e0325574. doi: 10.1371/journal.pone.0325574 (PMC13056177; doi:10.1371/journal.pone.0325574)
Supplement: Attachment — Submitted filename: CVM_mucus_PLOS_rebuttal.docx [file pone.0325574.s004.docx]

Reviewer #1: The paper is well written how ever few questions can improve the manuscript more

• **What are the possible problems with using cervical mucus from dead macaques, and how might it be different from mucus taken from live animals during their normal cycle?**

The mucus is removed from macaques at the time of necropsy (time of death). In other words, this is more or less a “fresh collection," and we do not expect any differences compared to those taken from an anesthetized live animal.

• **Is HEPES buffer a good choice to compare with cervical mucus? Why or why not — does it match the conditions inside the body?**

HEPES was chosen because it is a proven (and ideal) buffer for sperm. Our assisted reproductive core (ART core) has optimized sperm buffer based in HEPES for a long time with an extensive publication record and is HEPES is also one of several recommended buffers by andrology guidelines. We added references in our revision. Conceptually, as for a comparator, our focus here is on the viscosity of solution. In this respect, HEPES is like any simple pH-buffering ionic solution in that the viscosity is very low and similar to water (see reference below)>

WHO Laboratory Manual for the Examination and Processing of Human Semen. 6th ed. Geneva: World Health Organization; 2010.

• **How could this study help people with fertility problems? What might make it hard to use these results in real life?**

Thank you for this question. The thick impenetrable mucus secreted throughout most of the cycle restricts sperm entry into the upper tract for only a few days prior to ovulation. Women who fail to display ovulatory mucus at the time of ovulation carry a diagnosis of ‘cervical factor’ infertility. It is estimated that 5–20% of female infertility results from a ‘cervical factor’. This type of infertility has largely been ignored due to modern infertility treatments like intrauterine insemination allowing clinicians to bypass the cervix with a catheter and the lack of objective tools for measuring mucus changes and correlating them to changes in fertility (ie. effects on sperm). Thus, our study adds a few things to this field: First, it uses an objective measure of viscoelasticity (PTMR), a first in this field as far as we know, to assess cervical mucus samples and this could be used as tool for diagnosis and evaluation of treatment in the future. Second, we directly demonstrate a connection between viscosity of mucus and sperm velocity. While this hypothesized, this direct translational experiment has also not been done and again, further supports the translatability of PTMR measurements. Finally, our use of rhesus also supports the NHP model as platform future studies could be conducted to study interventions (both infertility and contraceptive) targeting the endocervix.

• **Why did the scientists use log values (log10) instead of the original numbers for speed and thicknes.**

We used a logarithmic representation of the values as it more clearly shows the correlation between speed and viscosity. However, this representation does not alter the underlying values of the data. We suggest that this representation is more easily interpreted than similar presentations using different rheological techniques such as Figure 4 in reference 6 of the manuscript (below).

Wolf, D. P., Blasco, L., Khan, M. A. & Litt, M. Human cervical mucus. IV. Viscoelasticity and sperm penetrability during the ovulatory menstrual cycle. *Fertil Steril* **30**, 163–169 (1978).

• **could things like pH or chemicals in the mucus affect sperm speed**

Yes. Certainly. For this reason we choose a sperm optimized HEPES based buffer (see prior question)

**Reviewer #2: The manuscript explores the relationship between cervical mucus viscoelasticity and sperm velocity using particle tracking microrheology (PTMR) in rhesus macaques. The current study lacks methodological depth. The data appear sparse. As such, the manuscript falls short of the level of detail and rigor expected for publication. The use of rhesus macaques despite being euthanized for unrelated reasons could have influenced rheological properties is not justified, especially since the conclusions are framed in a human reproductive context. Human cervical mucus and sperm are relatively easy to obtain for in vitro studies across the menstrual cycle.**

Thank you. As the reviewer notes, human mucus samples can be obtained as we’ve published extensively using clinical trial examining mucus changes. However, this is a translational paper interested in both assessing a novel measure of mucus viscoelastic changes and its correlation with sperm movement. A non human primate model presents a strong and validated experimental platform (see reference below) for these of studies. And while human clinical trials can be a resource for specimen collection, there are also practical considerations that led us to choose NHP mucus, with the most relevant being, the laboratory we conducted these studies takes place within the Oregon National Primate Research Center and we have access to ample fresh NHP specimens and animals. We acknowledge the limitations of translational studies in our limitations section and the usual need for validation studies. We took care not over-extrapolate our findings and keep manuscript length tight to reflect the data presented.

[1]

Han L, Park D, Reddy A, Wilmarth PA, Jensen JT. Comparing endocervical mucus proteome of humans and rhesus macaques. PROTEOMICS – Clinical Applications 2021;15:2100023. https://doi.org/10.1002/prca.202100023

**Reviewer #3: This is an automated report for PONE-D-25-26377. This report was solicited by the PLOS One editorial team and provided by ScreenIT.**

**ScreenIT is an independent group of scientists developing automated tools that analyze academic papers. A set of automated tools screened your submitted manuscript and provided the report below. Each tool was created by your academic colleagues with the goal of helping authors. The tools look for factors that are important for transparency, rigor and reproducibility, and we hope that the report might help you to improve reporting in your manuscript. Within the report you will find links to more information about the items that the tools check. These links include helpful papers, websites, or videos that explain why the item is important. While our screening tools aim to improve and maintain quality standards they may, on occasion, miss nuances specific to your study type or flag something incorrectly. Each tool has limitations that are described on the ScreenIT website. The tools screen the main file for the paper; they are not able to screen supplements stored in separate files. Please note that the Academic Editor had access to these comments while making a decision on your manuscript. The Academic Editor may ask that issues flagged in this report be addressed. If you would like to provide feedback on the ScreenIT tool, please email the team at ScreenIt@bih-charite.de. If you have questions or concerns about the review process, please contact the PLOS One office at plosone@plos.org.**
