# Supplementary material for: Cervical mucus viscoelasticity and sperm velocity are correlated and concentration-dependent in Vitro
Source: PLoS One. 2026 Apr 7;21(4):e0325574. doi: 10.1371/journal.pone.0325574 (PMC13056177; doi:10.1371/journal.pone.0325574)
Supplement: Attachment — Submitted filename: CVM_mucus_PLOS_rebuttal_2_final_clean.docx [file pone.0325574.s005.docx]

We thank the reviewers for the careful and thoughtful evaluation of our manuscript. We appreciate the constructive suggestions, which have improved the clarity, focus, and methodological transparency of the work. Below we provide detailed responses and indicate the revisions made in the manuscript.

**Reviewer #1: No Comments**

**Reviewer #4: This is in general a well-written manuscript providing new and interesting information. The manuscript sometimes lacks focus and should be improved taking this criticism into account. Some examples are given below.
The authors have also provided satisfying responses to the reviewers’ questions, but they should have added respective changes and explanations to the manuscript. This should be rectified immediately.**

We thank the reviewers for their insights and noted that they were not fully satisfied with the data availability. This has been remedied by adding helpful analysis code and all data for each female animal (n=6) to at GitHub repository (<https://github.com/MMarkovetz/CVM_Paper_Data.git>, see page 6, line 5 in revised manuscript).

**In addition, the following concerns must be addressed:
(It is always advisable to provide line numbers for a submitted manuscript to allow for easier review).
In the Material and Methods section, the authors must provide information on the number of semen donors and ejaculates included in the investigation. How was this considered in the statistical analysis. There is also no information if and how semen characteristics were determined. Were there any inclusion/exclusion criteria taken into account?**

Semen used for these experiments was obtained from multiple adult male rhesus macaques during routine collection sessions at the Oregon National Primate Research Center. Across the experimental period, several fresh ejaculates from at least three individual males were available and used in these studies. Specifically, samples included in this work exhibited at least approximately 50 x 10⁶ sperm/mL concentration and ≥65% total motility at baseline evaluation. For sperm tracking experiments, semen was diluted to a standardized concentration (1x 10⁶ sperm/mL) to ensure optimal tracking density within each imaging field. Because sperm were normalized to this working concentration prior to addition to mucus, baseline semen concentration was not incorporated as a variable in the statistical analysis. We have clarified this in the manuscript:

Page 4, Lines 19-23, New Text:

Ejaculates from at least three individual males were included. Only samples demonstrating ≥50 × 10⁶ sperm/mL and ≥60% total motility after liquefaction were used. For tracking experiments, sperm were diluted to 1 × 10⁶ sperm/mL prior to addition to mucus; therefore, baseline semen concentration was not included as a variable in statistical analyses.

**Cervical mucus was collected at different stages of the reproductive cycle. Please provide information at what stage of the cycle the samples included in the study were collected and if and how this affected viscosity. How were respective differences considered in the study?**

Regarding cervical mucus collection, samples were obtained at necropsy from adult female macaques at differing stages of the menstrual cycle, as determined by steroid hormone levels at time of collection. Because of the limited number of animals per cycle stage, the study was not powered to compare rheological properties across specific hormonal phases. Importantly, the central experimental design relied on serial dilution within each individual mucus sample. Thus, each animal served as its own internal comparator, allowing evaluation of concentration-dependent changes independent of baseline cycle stage. We have clarified this in the revised manuscript.

Page 4, Lines 8-11, New text:

The study was not powered to compare specific cycle phases; therefore, analyses focused on serial dilution within each sample, with each animal serving as its own internal control for concentration-dependent effects.

**“Sperm were labeled with Hoescht…” Please give the respective number and information on the Hoechst staining and correct the name.**

We have corrected the spelling of the fluorescent dye to Hoechst 33342 and now provide the working concentration and incubation conditions used for sperm labeling. These details have been added to the Materials and Methods section.

Page 6, Line 7-8, New text:

Sperm were fluorescently labeled with Hoechst 33342 (2 µg/mL) for 10 minutes at 37 °C to improve trackability.

**“Sperm motion was tracked using a slightly modified version of the PTMR program”. Provide details on the modifications as well as settings used for sperm velocity analysis (e.g. frame settings etc). At what concentration were sperm added to the mucus samples?**

The only modifications were to the shape parameters used to identify sperm heads. Code parameter bead_size was increased from 11 to 17, and eccentricity was increased from 0.3 to 0.5, but the underlying algorithm was unaltered. These additional details are now included in the manuscript

Page 6, Line 12-14, New text:

The only modifications were to the shape parameters used to identify sperm heads. Code parameter bead_size was increased from 11 to 17, and eccentricity was increased from 0.3 to 0.5, but the underlying algorithm was unaltered.

**What was the pH and osmolality of the (diluted) mucus samples? It is ok that Hepes was used to buffer pH but the pH is not given anywhere. Changes in osmolality will influence sperm velocity.**

We acknowledge the reviewer’s point regarding pH and osmolality. Due to the very small sample volumes available (5–15 µL per condition), direct measurement of these parameters was not feasible. However we have previously shown that mucus is a strong buffer on its own (Hill et al., Eur. Resp. J., 2018), so we do not anticipate much pH change across samples. Osmolality correlates directly with concentration, so we would expect a proportional decrease in osmolality as samples were diluted. We are unable here to assess how osmolality might have effected the sperm, but we are also unaware of inert reagents that we could add to the buffer to modify osmolality without also affecting sperm physiology. We have added a clarifying statement in the Discussion acknowledging this limitation.

Page 10, Line 2-6, New text:

However, there are limitations to this methodology as well. For example, we must assume that the strong buffer capacity of the mucus itself limits pH changes (Hill et al., ERJ, 2018). Additionally, osmolality affects sperm behavior, but are unaware of inert reagents that could modify only buffer osmolality without affecting sperm physiology.

**Information on statistical analysis is not detailed enough.**

We thank the reviewer and have added more detail regarding statistical analyses performed. The Statistical Analysis section has been expanded for clarity. We now explicitly state that mean values per dilution per sample were used to determine Spearman correlation coefficient, r, and associated p-values using MATLAB, with statistical significance defined as α = 0.05.

Page 6, Line 20, 22-23. New text:

“… using a Pearson correlation test.”

“Values reported are ensemble mean values unless otherwise noted. Significance for all tests was determined using an α value of 0.05.”

**Some statements in the results section should be moved to the discussion, e.g. “Velocity was highly variable within each sample, illustrating the heterogeneity of both mucus biophysics and sperm motility” and “…, which reflects the known phenomenon that cellular swimmers can be faster in viscoelastic media compared to purely viscous media”. These statements also require additional discussion/interpretation.**

We agree that several interpretive statements in the Results section were more appropriate for the Discussion. Specifically, commentary regarding heterogeneity of sperm velocity within samples and the phenomenon of enhanced swimming speed in viscoelastic media has been relocated to the Discussion and expanded with additional context and citation. We have also removed the final interpretive sentence from the Results section as suggested.

Page 9-10, Line 24-1.

**The last sentence of the results section (“These results imply that…”) is also an interpretation that is already covered in the discussion. The sentence should be deleted here.**

We appreciate the suggestion and have removed this sentence.

**Discussion**

**I agree with the statement “that PTMR may be used as a surrogate marker of sperm motility… and indicator of the fertility status of the mucus”. This is an interesting and important result. You should consider that upon reversion, PTMR may also be an interesting test for sperm function in men with fertility problems. Please consider this aspect in the discussion.**

We thank the reviewer for this keen insight. We have now added discussion of how PTMR-based approaches could complement existing Computer Assisted Sperm Analysis (CASA) methodologies, particularly in evaluating sperm function under physiologically relevant viscoelastic conditions. This may be of relevance in cases of unexplained male infertility.

Page 10, Line 2, New text:

Furthermore, our technique may represent a robust alternative to existing CASA methodologies.

**It is recommended to reduce the paragraph on page 17 (“Our studies may also be useful in understanding fertility changes… cervical mucus hydration as fertility regulator.”) because these aspects were not investigated in this study.**

Thank you, in response to the recommendation to reduce the paragraph discussing broader fertility changes and cystic fibrosis, we have substantially shortened this section to maintain focus on findings directly supported by the present data.

Page 11, Line 4-6, New text:

Small molecule drugs that modulate mucus viscosity via ion channels present in both the endocervix and other organ systems are one set of likely drug targets.

**Also, Figure 4 presents an interesting concept, but the contents were not addressed in the present investigation. It should be considered to remove it.**

Thank you. we agree that Figure 4 presents a conceptual framework not directly tested in this study. To maintain focus and avoid overextension of the conclusions, we have removed Figure 4 associate text from the revised manuscript.

**In scientific writing it is improper grammar to begin a sentence with an abbreviation. Please correct this throughout the manuscript.**

We have made these corrections where appropriate throughout the manuscript.

**All figures and tables should “stand alone”, i.e. their legends should provide complete descriptions on what is being presented so that the reader does not have to refer to the manuscript text. Where appropriate, the legend must include information on the species, numbers of individuals or repetitions.**

Finally, we have carefully edited all figure legends so that they stand alone. Legends now specify species (Macaca mulatta), number of animals, number of technical replicates, dilution ranges, and statistical approaches where appropriate.
